# 4′-Fluorouridine inhibits alphavirus replication and infection *in vitro* and *in vivo*

**DOI:** 10.1128/mbio.00420-24

**Published:** 2024-05-03

**Authors:** Peiqi Yin, Nicholas A. May, Laura Sandra Lello, Atef Fayed, M. Guston Parks, Adam M. Drobish, Sainan Wang, Meghan Andrews, Zachary Sticher, Alexander A. Kolykhalov, Michael G. Natchus, George R. Painter, Andres Merits, Margaret Kielian, Thomas E. Morrison

**Affiliations:** 1 Department of Cell Biology, Albert Einstein College of Medicine, Bronx, New York, USA; 2 Department of Immunology and Microbiology, University of Colorado School of Medicine, Aurora, Colorado, USA; 3 Institute of Bioengineering, University of Tartu, Tartu, Estonia; 4 Emory Institute for Drug Development (EIDD), Atlanta, Georgia, USA; 5 Drug Innovations Ventures at Emory (DRIVE), Atlanta, Georgia, USA; 6 Department of Pharmacology and Chemical Biology, Emory University School of Medicine, Atlanta, Georgia, USA; Columbia University Medical College, New York, New York, USA

**Keywords:** alphavirus, chikungunya virus, mayaro virus, antiviral, RNA replication

## Abstract

Chikungunya virus (CHIKV) is an enveloped, positive-sense RNA virus that has re-emerged to cause millions of human infections worldwide. In humans, acute CHIKV infection causes fever and severe muscle and joint pain. Chronic and debilitating arthritis and joint pain can persist for months to years. To date, there are no approved antivirals against CHIKV. Recently, the ribonucleoside analog 4′-fluorouridine (4′-FlU) was reported as a highly potent orally available inhibitor of SARS-CoV-2, respiratory syncytial virus, and influenza virus replication. In this study, we assessed 4′-FlU’s potency and breadth of inhibition against a panel of alphaviruses including CHIKV, and found that it broadly suppressed alphavirus production in cell culture. 4′-FlU acted on the viral RNA replication step, and the first 4 hours post-infection were the critical time for its antiviral effect. *In vitro* replication assays identified nsP4 as the target of inhibition. *In vivo*, treatment with 4′-FlU reduced disease signs, inflammatory responses, and viral tissue burden in mouse models of CHIKV and Mayaro virus infection. Treatment initiated at 2 hours post-infection was most effective; however, treatment initiated as late as 24–48 hours post-infection produced measurable antiviral effects in the CHIKV mouse model. 4′-FlU showed effective oral delivery in our mouse model and resulted in the accumulation of both 4′-FlU and its bioactive triphosphate form in tissues relevant to arthritogenic alphavirus pathogenesis. Together, our data indicate that 4′-FlU inhibits CHIKV infection *in vitro* and *in vivo* and is a promising oral therapeutic candidate against CHIKV infection.

**IMPORTANCE:**

Alphaviruses including chikungunya virus (CHIKV) are mosquito-borne positive-strand RNA viruses that can cause various diseases in humans. Although compounds that inhibit CHIKV and other alphaviruses have been identified *in vitro*, there are no licensed antivirals against CHIKV. Here, we investigated a ribonucleoside analog, 4′-fluorouridine (4′-FlU), and demonstrated that it inhibited infectious virus production by several alphaviruses *in vitro* and reduced virus burden in mouse models of CHIKV and Mayaro virus infection. Our studies also indicated that 4′-FlU treatment reduced CHIKV-induced footpad swelling and reduced the production of pro-inflammatory cytokines. Inhibition in the mouse model correlated with effective oral delivery of 4′-FlU and accumulation of both 4′-FlU and its bioactive form in relevant tissues. In summary, 4′-FlU exhibits potential as a novel anti-alphavirus agent targeting the replication of viral RNA.

## INTRODUCTION

Chikungunya virus (CHIKV) is a mosquito-transmitted positive-strand RNA virus from the *Alphavirus* genus in the *Togaviridae* family (1). This genus includes significant human pathogens such as CHIKV, Mayaro virus (MAYV), o’nyong-nyong virus (ONNV), Ross River virus (RRV), and the Eastern, Western, and Venezuelan equine encephalitis viruses, as well as less pathogenic viruses such as Sindbis virus (SINV) and Semliki Forest virus (SFV) (2). In humans, acute CHIKV infection causes chikungunya fever with symptoms including fever, rash, and severe joint pain (3, 4). This acute infection can progress to chronic debilitating polyarthritis in a significant proportion of patients, with symptoms lasting for months to years (5). In recent years, CHIKV has spread globally and imposed a significant health burden on millions of people (6, 7). The dramatic expansion of CHIKV infection reflects both the increased prevalence of its mosquito vectors, driven in part by global warming, and the generation of viral mutations that promote more effective transmission (6–8). To date, there are no licensed antivirals against CHIKV or other alphaviruses, although several candidate CHIKV vaccines are in development and one was recently approved for human use (9).

CHIKV is an enveloped, positive-sense RNA virus with a ~12 kb genomic RNA that includes a 5′ N7-methylguanosine cap, a 3′ polyA tail, and two open reading frames (ORFs) (1). The first ORF encodes a non-structural (ns) polyprotein that is processed to produce the four ns proteins (nsP1-4) that mediate genome replication (10). The second ORF, situated downstream of a subgenomic promoter, encodes the viral structural proteins capsid-E3-E2-6K/TF-E1 (10, 11). Following virus entry and membrane fusion, the viral genomic RNA is released into the cytoplasm and translated to produce the ns polyprotein, cleavage products of which form the early replication complex. This synthesizes a negative-sense RNA, which forms a double-stranded RNA (dsRNA) intermediate with the genomic strand and is then used as a template to generate both positive-sense genomic and subgenomic RNAs. The subgenomic RNA is translated to produce the structural proteins, which package the genomic RNA (12, 13) and assemble progeny virus particles (1, 11).

Alphavirus RNA replication occurs within membrane invaginations known as spherules, which create a favorable environment for viral RNA synthesis and appear to protect the viral dsRNA intermediates from recognition by host sensors (14). Spherules contain the replication complex assembled by the nsPs and the dsRNA intermediate (15, 16). Production of viral RNA within spherules depends on the unique enzymatic and non-enzymatic functions of the nsPs, as briefly summarized here and reviewed in (10, 17). nsP1 is the viral RNA capping enzyme and the membrane anchor of the replication complex. nsP2 contains an N-terminal RNA helicase/NTPase region and a C-terminal cysteine protease that mediates the essential regulated cleavage reactions of the ns polyprotein. nsP3 contains a macrodomain, a central zinc-binding domain, and a disordered region that mediates host factor interactions. Lastly, nsP4 is the viral RNA-dependent RNA polymerase (RdRp).

There is a critical need for antiviral strategies against alphavirus infections. Antibodies targeting the alphavirus envelope proteins can be highly potent inhibitors of infection (18), but antibody production tends to be costly and delivery by infusion can be cumbersome. Direct-acting antiviral therapies that could be orally administered would be an important advance, particularly given the prevalence of CHIKV infection in resource-poor countries. A number of alphaviral targets for such therapies are under investigation, as reviewed in references 19–23. For example, the identification of alphavirus receptors and definition of the structures of receptor-virus complexes (reviewed in reference 24) opens the door to targeting these interactions with small molecules. The capsid protein has a hydrophobic pocket that interacts with the E2 endodomain during budding, and also a critical autoprotease activity, both of which have been investigated as targets for small molecules (25, 26). Other antiviral strategies have concentrated on the enzymatic activity of the nsPs, particularly the nsP2 protease activity and the RdRp activity of nsP4.

nsP4 is the most highly conserved protein among alphaviruses (23). Several nucleoside analogs have been shown to inhibit the replication cycles of CHIKV and other alphaviruses. Favipiravir and its defluorinated analog inhibit *in vitro* replication of various CHIKV strains and other arthritogenic alphaviruses (27). β-D-N4-hydroxycytidine (NHC) exhibits more potent anti-CHIKV effects in cell culture compared to favipiravir and ribavirin (28, 29). Sofosbuvir, an FDA-approved drug against hepatitis C virus, demonstrated significant activity against CHIKV (30), while a compound related to benzimidazole can inhibit infection by several CHIKV strains and SINV (31).

**Fig 1:**
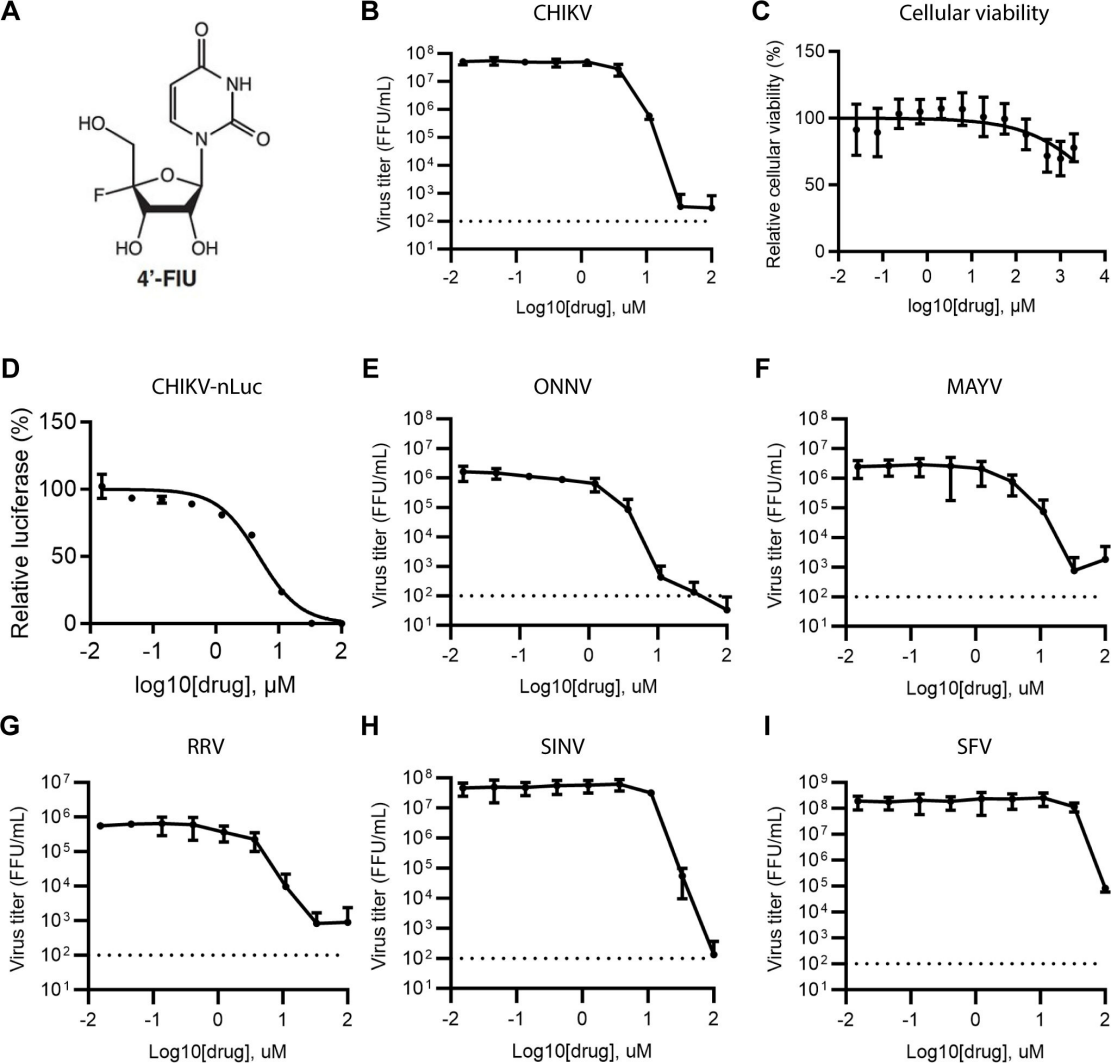
Dose-response studies of 4′-FlU in alphavirus-infected U-2 OS cells. (**A**) Structure of 4′-FlU. (**B**) Effect of 4′-FlU on CHIKV production. U-2 OS cells were infected with CHIKV at a multiplicity of infection = 0.1 for 1 h and then treated with the indicated concentrations of 4′-FlU. Media were harvested at 24 hpi and titered by focus forming assay on U-2 OS cells. The data shown represent the mean and standard deviation of three independent experiments. (**C**) U-2 OS cells were incubated with the indicated concentrations of 4′-FlU for 24 h. Cell viability was assessed using PrestoBlue and normalized to control DMSO-treated cells. The data shown represent the mean ± SD of three independent experiments. (**D**) Effect of 4′-FlU on CHIKV-nLuc. U-2 OS cells were infected with CHIKV-nLuc and treated with 4′-FlU as in panel **B** or with DMSO (vehicle control). Luciferase activity was measured at 24 hpi and normalized to that of control cells. Data points represent three independent repeats. (**E–I**) Effect of 4′-FlU on the production of various alphaviruses. U-2 OS cells were infected with ONNV (**E**), MAYV (**F**), RRV (**G**), SINV (**H**), or SFV (**I**) and the effect of 4′-FlU determined as in panel **B**. The data shown represent the mean and standard deviation of three independent experiments. EC_50_ and CC_50_ values from this figure are summarized in Table 1.

Recently 4′-fluorouridine (4′-FlU, EIDD-2749) (Fig. 1A) was reported to be a highly potent inhibitor of SARS-CoV-2 and of viruses of the *Mononegavirales* order, with oral efficacy in animal models of infection by SARS-CoV-2, respiratory syncytial virus (RSV), and seasonal and highly pathogenic influenza viruses (32, 33). Here we demonstrated that treatment with 4′-FlU broadly suppressed the production of alphaviruses including CHIKV in cell culture. Time-of-addition studies showed that the first 4 hours post-infection (hpi) were the critical time for the antiviral effect. Using CHIKV replication assays, we defined inhibition of RNA replication by nsP4 as the mechanism of action of 4′-FlU. We also determined the oral efficacy of 4′-FlU in mouse models and found that treatment with 4′-FlU reduced virus burden and inflammation in CHIKV- and MAYV-infected mice.

## RESULTS

### 4′-FlU broadly suppresses alphavirus infection

We first evaluated the antiviral potency of 4′-FlU against CHIKV using the CHIKV strain 181/25, an attenuated derivative of Southeast Asian human isolate strain AF15561 (34). U-2 OS cells were infected for 1 hour (h) with CHIKV at a multiplicity of infection (MOI) of 0.1 focus forming units (FFU) per cell. The cells were then incubated with various concentrations of 4′-FlU and the media were collected at 24 hpi and titered by focus forming assay (FFA) on U-2 OS cells. 4′-FlU strongly inhibited CHIKV production, reducing viral titers by ~5 logs at a concentration of 33 µM, with a 50% effective concentration (EC_50_) of 3.89 µM (Fig. 1B; Table 1). Prior studies demonstrated that treatment with up to 500 µM 4′-FlU is not cytotoxic to several human and animal cell lines (HEp-2, MDCK, BHK-T7, and BEAS-2B) (32). Analysis of 4′-FlU cytotoxicity in U-2 OS cells showed that the half-maximal cytotoxic concentration (CC_50_) was greater than 2,000 µM, resulting in a high selectivity index (SI = CC_50_/EC_50_) of >514 (Fig. 1C). We also tested 4′-FlU against a CHIKV 181/25 reporter virus that encodes nanoluciferase (nLuc) downstream of the viral subgenomic promoter. 4′-FlU inhibited nLuc production with an EC_50_ of 4.85 µM, similar to the EC_50_ observed in the virus production assay (Fig. 1D). To determine the breadth of antiviral activity of 4′-FlU against alphaviruses, we assessed its inhibitory activity against a panel of re-emerging alphaviruses (ONNV, MAYV, and RRV) and less pathogenic alphaviruses (SINV and SFV). 4′-FlU strongly inhibited MAYV, ONNV, and RRV production, with similar EC_50_ values as those observed for CHIKV (Fig. 1E through G; Table 1). Production of SINV and SFV was also inhibited by 4′-FlU but with ~3-fold to 10-fold higher EC_50_ values (Fig. 1H and I; Table 1).

**TABLE 1:** Summary of 4′-FlU inhibition of alphavirus infection^*a*^

Virus or replicon	EC_50_ (μM)	95% CI (μM)	Cell line	Assay readout
CHIKV 181/25	3.89	2.71 to 5.49	U-2 OS	Virus titration
CHIKV-nLuc 181/25	4.85	4.05 to 5.75	U-2 OS	Luciferase reporter
CHIKV-nLuc-replicon	5.27	4.04 to 6.90	U-2 OS	Luciferase reporter
ONNV	1.19	0.68 to 1.99	U-2 OS	Virus titration
MAYV	2.77	1.65 to 4.55	U-2 OS	Virus titration
RRV	3.73	1.42 to 9.83	U-2 OS	Virus titration
SINV	~12.40	(Very wide)	U-2 OS	Virus titration
SFV	~35.12	(Very wide)	U-2 OS	Virus titration

^*a*^ The CC_50_ in U-2 OS cells was >2,000 μM, the highest concentration tested.

### 4′-FIU acts during the initial 4 hours of the CHIKV life cycle

**Fig 2:**
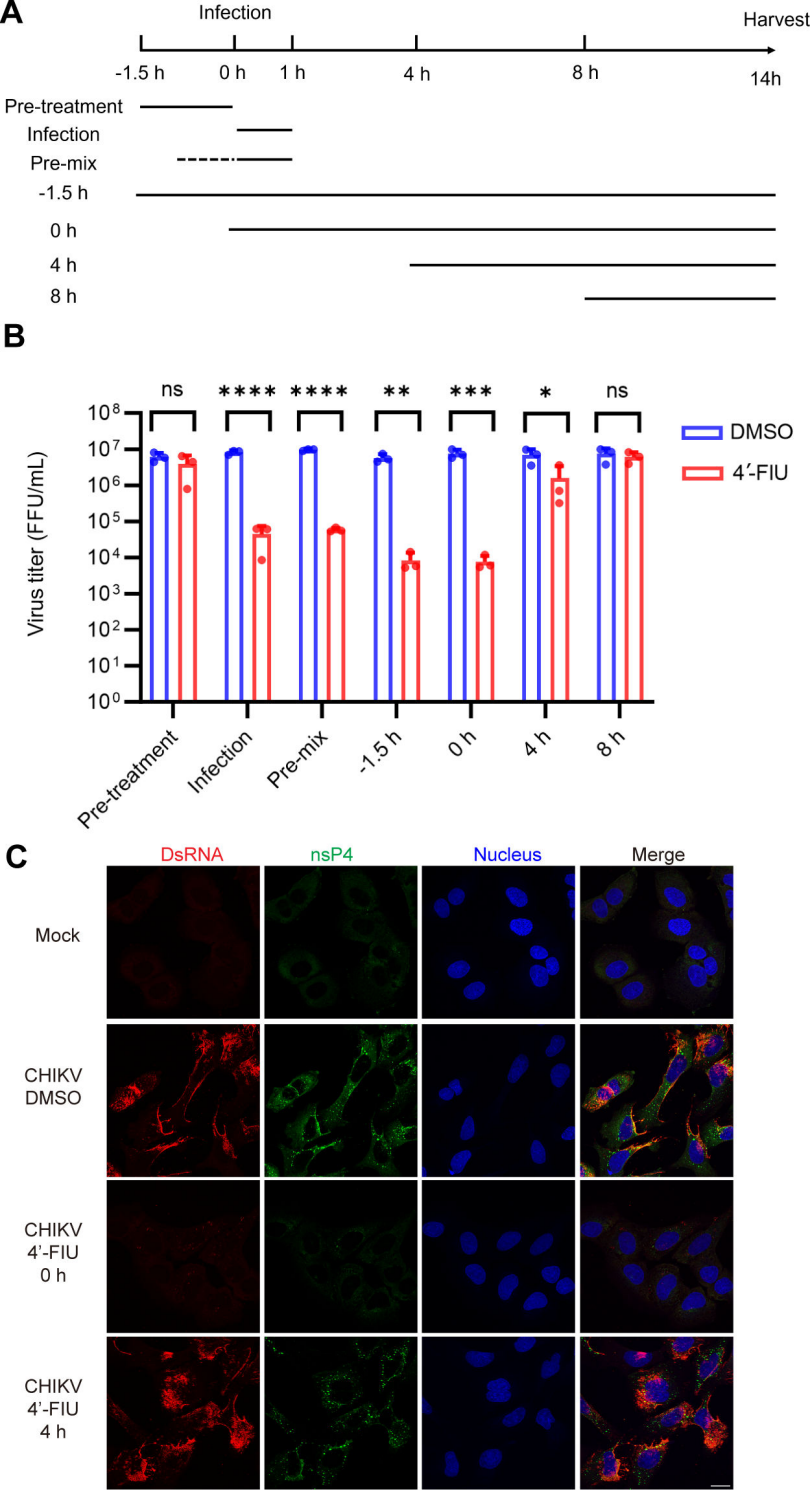
Time of addition studies with 4′-FlU. (**A**) Schematic of time of addition experiment. (**B**) U-2 OS cells were infected with CHIKV at MOI = 3 for 1 h. 10 µM 4′-FlU was added as indicated. The media were harvested at 14 hpi and titered by FFA. Mean  ±  SD of three independent experiments (shown as points). Statistical significance was calculated by two-way ANOVA with Sidak’s multiple comparisons test. *P < 0.05, **P < 0.01, ***P < 0.001, ****P < 0.0001, ns not significant. (**C**) U-2 OS cells were infected with CHIKV at MOI = 3 for 1 hour, and treated with 10 µM 4′-FlU at 0 hpi or four hpi. The cells were then fixed at 8 hpi, permeabilized, and immunostained with antibodies against dsRNA (red) and nsP4 (green). Figure shows representative images from two independent experiments. Scale bar = 20  µm.

To obtain insight into the mechanism of action of 4′-FlU, the compound was added at 10 µM at different times during CHIKV infection, and effects on virus production at 14 hpi were quantitated by comparison with cells treated with vehicle alone (Fig. 2A and B). This experiment was performed at an MOI of 3 to promote synchronized infection. Pre-treatment of the cells with 4′-FlU for 1.5 h prior to infection did not produce inhibition, suggesting that 4′-FlU does not limit virus production by targeting host processes. We compared inhibition when 4′-FlU was pre-incubated with CHIKV particles for 1 hour vs when CHIKV and 4′-FlU were directly added to cells without virus pre-incubation, with infection in both cases continued in the presence of the drug for 1 hour. Both treatments reduced virus production by ~2 logs, suggesting that 4′-FlU does not alter or damage CHIKV particles. 4′-FlU reduced CHIKV titers by ~3 logs when added to the cells either 1.5 h prior to or at the same time as virus addition, followed by a 14-hour infection in the presence of the drug (−1.5 hours and 0 hour). Inhibition of virus production was dramatically reduced when 4′-FlU was added to cells at 4 hpi, and essentially no decrease in virus production was observed when 4′-FlU was added at 8 hpi. Cells were also infected with CHIKV for 1 h, 4′-FlU added at 0 or 4 hpi, and the cells stained at 8 hpi with antibodies to dsRNA and nsP4 (Fig. 2C). Inhibition of dsRNA and nsP4 production was observed when the drug was added at 0 hpi but not at 4 hpi. Together, our data indicate that the initial 4 hpi are the crucial period for the antiviral impact of 4′-FlU, in keeping with the time course of alphavirus RNA replication (10).

### Alphavirus RNA replication is inhibited by 4′-FlU

**Fig 3:**
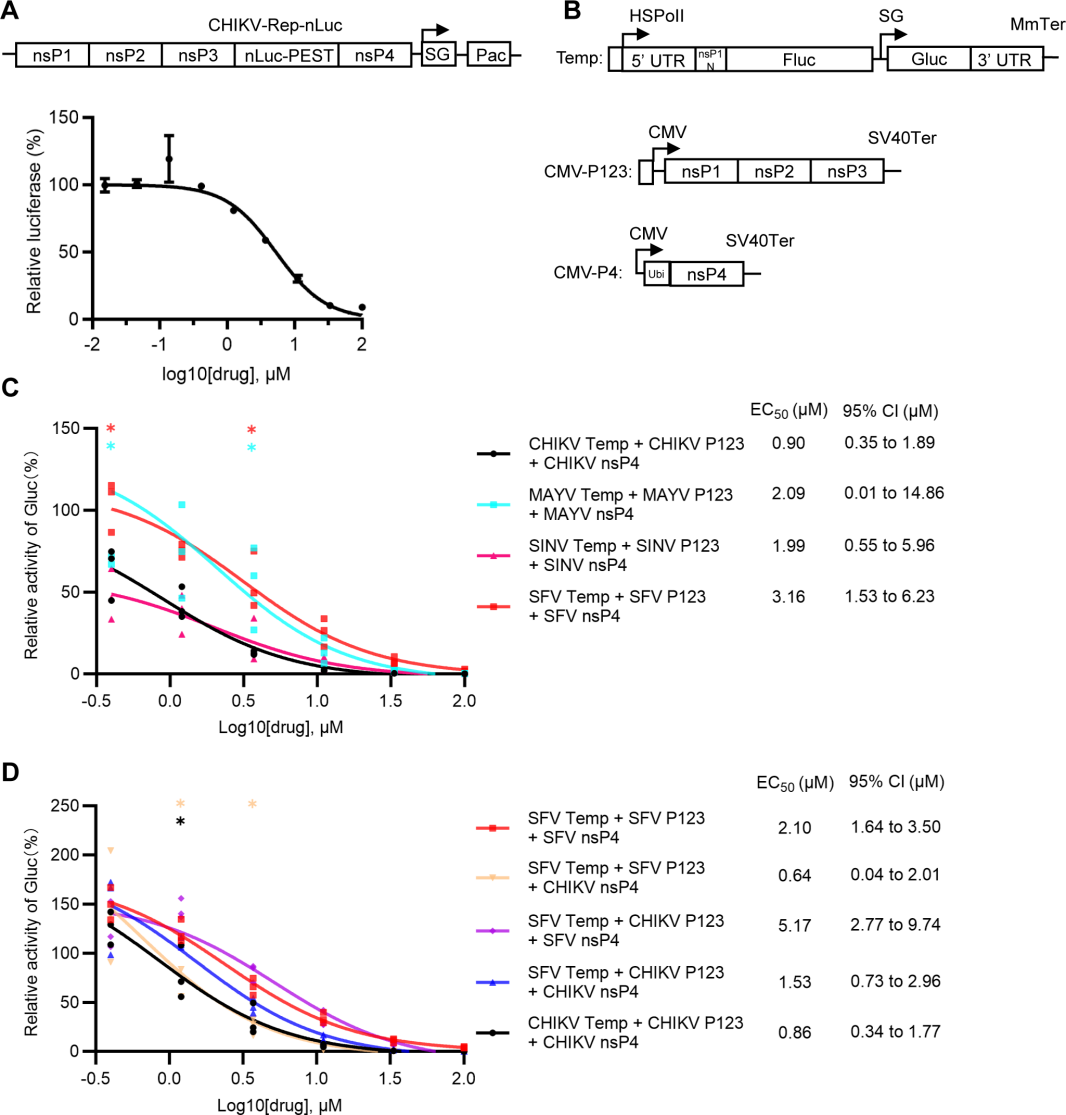
4′-FlU blocks alphavirus RNA replication. (**A**) Effect of 4′-FIU on CHIKV replicon. The design of the CHIKV-Rep-nLuc replicon is shown in the diagram. SG, subgenomic promoter; Pac, puromycin N-acetyltransferase (selection marker). A stable U-2 OS cell line harboring this replicon was treated with the indicated concentrations of 4′-FlU for 24 hours. Luciferase activity was determined and normalized to the DMSO-treated control. Data show the mean and standard deviation of three independent experiments. (**B**) Diagram of expression plasmids for the *trans*-replicase assay, showing template RNA (top) and replicase protein expression constructs (CMV-P123 and CMV-P4). Labels indicate HSPolI, a truncated promoter (residues −211 to –1) for human RNA polymerase I; MmTer, a terminator for RNA polymerase I in mice; CMV, immediate early promoter of human cytomegalovirus; SV40Ter, simian virus 40 late polyadenylation region. (**C**) Dose-response studies of 4′-FlU in alphavirus *trans*-replicase assays. U-2 OS cells were transfected with alphavirus *trans*-replicase constructs for 4 hours and treated with indicated concentrations of 4′-FlU for 20 hours. Gluc activity was determined and normalized to that of DMSO-treated controls. (**D**) Dose-response studies of 4′-FlU in mixed alphavirus *trans*-replicase assays. U-2 OS cells were transfected with the indicated constructs and Gluc activity was measured as in panel **C**. (**C and D**) Data points represent three independent experiments each performed with 2–3 technical repeats. Statistical significance was determined by two-way ANOVA with Sidak’s multiple comparisons test compared to “CHIKV Temp + CHIKV p123+ CHIKV nsP4” (**C**) or “SFV Temp + SFV p123+ SFV nsP4” (**D**). *P < 0.05, **P < 0.01.

To directly evaluate 4′-FlU for effects on alphavirus RNA replication, we first used a stable cell line harboring CHIKV-Rep-nLuc, a replicon that drives expression of a destabilized form of nLuc (Fig. 3A; Fig. S1A). The results showed that 4′-FlU inhibited the expression of the reporter with an EC_50_ of ~5.3 µM. We then tested the effect of 4′-FlU in a *trans*-replicase system in which co-expression of the precursor for nsP1-3 (P123) and nsP4 drive replication of a reporter RNA template (Fig. 3B; Fig. S1B). The results showed that 4′-FlU inhibited *trans*-replication for all of the alphavirus constructs tested (CHIKV, MAYV, SINV, and SFV) (Fig. 3C). Similar to the results of the virus production studies in Fig. 1, CHIKV replication was more sensitive to inhibition than SINV or SFV replication (EC_50_ of 0.9 µM vs. 2–3 µM). We took advantage of the modular nature of the *trans*-replicase system to test whether this reflected a difference in the 4′-FlU sensitivity of the nsP4 RdRp (Fig. 3D; Fig. S1C). The EC_50_ of 4′-FlU inhibition was reduced for the SFV replicase system when SFV nsP4 was substituted by CHIKV nsP4 (2.10 µM vs 0.64 µM). For the combination of CHIKV P123 and SFV nsP4, the EC_50_ was ~5.17 µM, while changing the nsP4 component to the CHIKV nsP4 produced an EC_50_ of 1.53 µM. Thus, sensitivity to 4′-FlU inhibition correlated with the source of the nsP4 in the *trans*-replicase assay. These results confirm that 4′-FlU acts on the alphavirus RdRp and indicate that the CHIKV RdRp is more sensitive to 4′-FlU than is the SFV RdRp.

### 4′-FIU treatment mitigates acute CHIKV infection and disease in C57BL/6 mice

**Fig 4:**
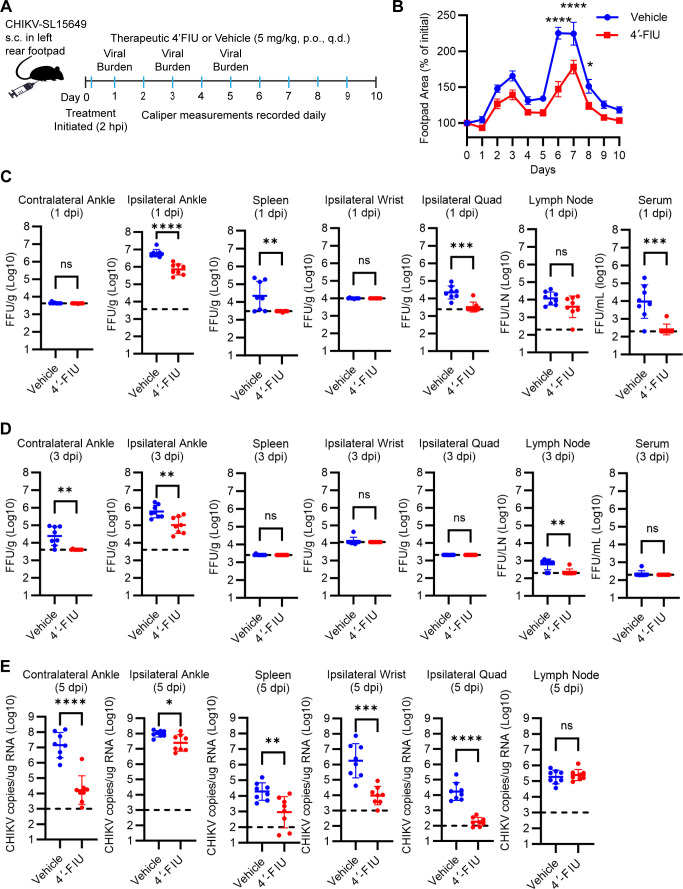
Oral treatment with 4′-FIU mitigates acute CHIKV infection and disease. (**A**) Schematic of experimental design created with BioRender.com. WT C57BL/6 mice (*n* = 8 mice/group) were inoculated with 10^3^ PFU of CHIKV in the left rear footpad. At 2 h post-inoculation, mice were treated by oral gavage with vehicle or 5 mg/kg 4′-FIU. Treatment continued once per day until the pre-determined experimental endpoints. (**B**) Swelling of the ipsilateral foot over time was measured by digital calipers. (**C-E**) At 1, 3, and 5 dpi, the viral burden in the tissues indicated was quantified by focus formation assay (**C and D**) or real-time quantitative polymerase chain reaction (**E**). Statistical significance was determined by repeated measures ANOVA with Tukey’s multiple comparison test (**B**) or Student’s unpaired *t*-test (**C-E**). *P < 0.05, **P < 0.01, ***P < 0.001, ****P < 0.0001.

To evaluate the antiviral efficacy of 4′-FIU against CHIKV infection *in vivo*, 4-week-old wild-type (WT) C57BL/6 mice (*N* = 8 mice/group) were inoculated with 10^3^ PFU of CHIKV SL15649 (35) in the left rear footpad (Fig. 4A). Two hours post-inoculation, mice were treated with 5 mg/kg of 4′-FIU or vehicle only (10 mM trisodium citrate in water) by oral gavage (p.o.). Treatment continued daily (q.d.). For all animal studies, mice were weighed daily as a sensitive indicator of health status. By this measure, and other unquantified observations (e.g., posture, gait, activity), we did not detect any evidence that 4′-FIU treatment negatively impacted the health of the animals. As observed in numerous prior studies (36–38), CHIKV infection following subcutaneous inoculation of WT C57BL/6 mice in the footpad leads to biphasic swelling of the ipsilateral foot and ankle, with an initial peak on days 2–3 post-infection and a second, more prominent peak on days 6–7 post-infection. 4′-FIU treatment did not produce significant inhibition of joint swelling at day 3 (Fig. 4B). However, the second phase of joint tissue swelling was reduced in magnitude in mice treated with 4′-FIU compared with control mice (Fig. 4B). For separate groups of mice, tissues were collected for viral burden analysis at 1-, 3-, and 5 days post-inoculation (dpi). At 1 dpi, after a single dose of 4′-FIU, the viral burden in the ipsilateral ankle, spleen, ipsilateral quad, and serum was reduced (Fig. 4C). At 3 dpi, the viral burden in the ipsilateral ankle, contralateral ankle, and draining popliteal lymph node was reduced in mice treated with 4′-FIU (Fig. 4D). At this time point, CHIKV typically has disseminated to the contralateral ankle, as seen in the control mice; 4′-FIU-treated mice did not have detectable infectious virus in this tissue. At 5 dpi, 4′-FIU-treated mice showed reduced CHIKV RNA levels in all tissues evaluated except the draining popliteal lymph node (Fig. 4E).

In parallel studies, biodistribution analyses were performed on tissues from mice that were dosed with 5 mg/kg 4′-FIU by oral gavage (Fig. S2). Tissues were harvested and the concentrations of 4′-FlU and 4′-FlU-5′-triphosphate (4′-FlU-5′-TP) were quantitated. The data demonstrate that 4′-FlU was rapidly absorbed into tissues and anabolized to the active 4′-FlU-5′-TP form at concentrations (e.g., ~1 nmol/g or higher for 4′-FlU-5′-TP) similar to those shown to be highly efficacious against influenza virus infection (33). There was minimal accumulation of 4′-FlU in all tested tissues over the 5-day course of treatment, while minimal accumulation of 4′-FlU-5′-TP in the spleen and left quad and no accumulation in the ankles and wrists was observed.

**Fig 5:**
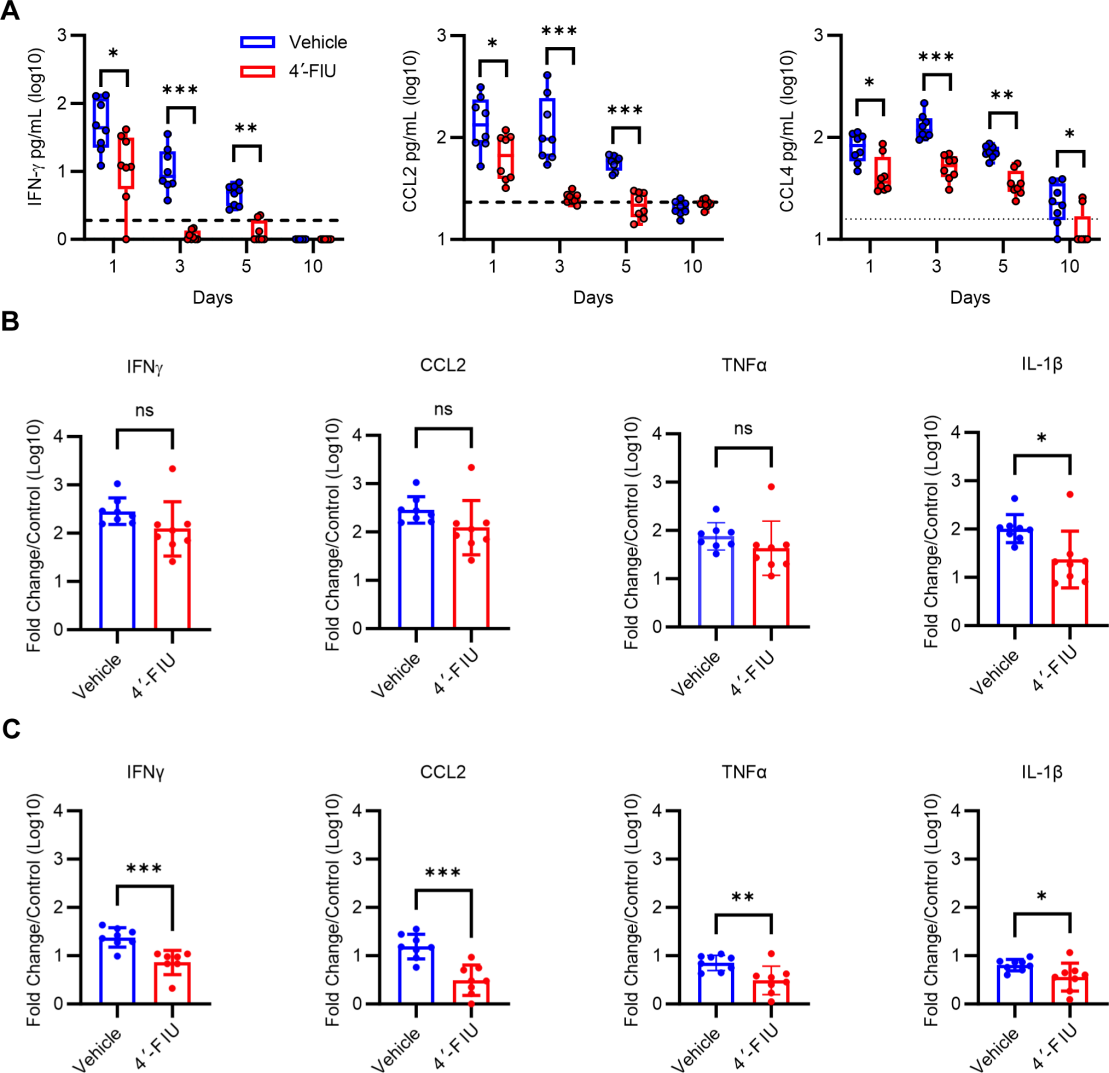
Oral treatment with 4′-FIU reduces CHIKV-induced inflammatory responses. (**A-C**) WT C57BL/6 mice were mock-inoculated (*n* = 4) or inoculated with 10^3^ PFU of CHIKV (*n* = 8 mice/group) in the left rear footpad. At 2 h post-inoculation, mice were treated by oral gavage with vehicle or 5 mg/kg 4′-FIU. Treatment continued once per day until the pre-determined experimental endpoints. (**A**) At 1, 3, 5, and 10 dpi, serum levels of the cytokines and chemokines indicated were quantified using an MSD U-plex assay. The dashed line indicates the baseline level of each protein detected in the serum of mock-inoculated control mice. (**B and C**) At 5 dpi, mRNA expression levels in the ipsilateral ankle (**B**) and contralateral ankle (**C**) for the genes indicated were quantified by RT-qPCR. Data are displayed as the fold change (log_10_) over the expression level detected in tissues of mock-inoculated control mice. Statistical significance was determined by Student’s unpaired student’s *t*-test. *P < 0.05, **P < 0.01, ***P < 0.001.

CHIKV infection elicits an inflammatory response that contributes to disease and tissue pathology (35, 36, 39–42). To investigate the impact of 4′-FIU treatment on the virus-induced inflammatory response, inflammatory cytokines, and chemokines were quantitated in sera collected at 1, 3, 5, and 10 dpi. As shown in Fig. 5A, the amount of IFN-γ, CCL2, and CCL4 in the circulation was reduced at 1, 3, and 5 dpi in 4′-FIU-treated mice compared with controls. To analyze the inflammatory response in joint-associated tissue, we performed real-time quantitative polymerase chain reaction (RT-qPCR) analysis of gene expression in tissues at 5 dpi. In the ipsilateral ankle, expression of IL-1β, but not other inflammatory genes, was reduced in 4′-FIU-treated mice (Fig. 5B). In the contralateral ankle, 4′-FIU treatment reduced expression of IFN-γ, TNF-α, IL-1β, and CCL2 (Fig. 5C), which is consistent with the reduced viral burden in the contralateral ankle at this time point (Fig. 4E). Collectively, these results demonstrate that oral treatment with 4′-FIU limits CHIKV infection and disease in mice.

### Oral treatment with 4′-FIU limits acute MAYV infection in C57BL/6 mice

**Fig 6:**
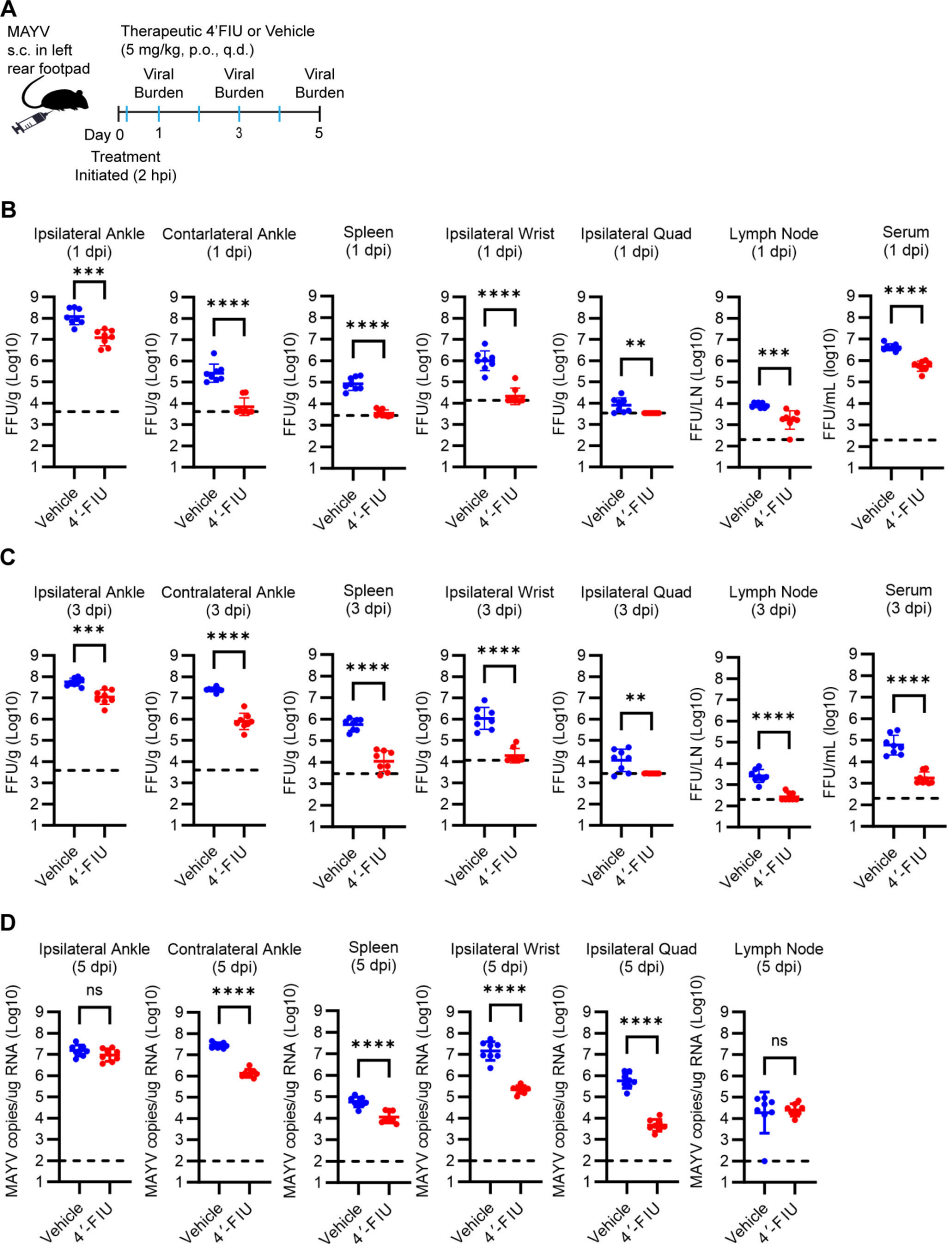
Oral treatment with 4′-FIU mitigates acute MAYV infection. (**A**) Schematic of experimental design generated with BioRender.com. WT C57BL/6 mice (*n* = 8 mice/group) were inoculated with 10^3^ PFU of MAYV in the left rear footpad. At 2 h post-virus inoculation, mice were treated by oral gavage with vehicle or 5 mg/kg 4′-FIU. Treatment continued once per day until the pre-determined experimental endpoints. (**B-D**) At 1, 3, and 5 dpi, the viral burden in the tissues indicated was quantified by focus formation assay (**B and C**) or RT-qPCR (**D**). Statistical significance was determined by unpaired Student’s *t*-test. **P < 0.01, ***P < 0.001, ****P < 0.0001.

To evaluate the antiviral breadth of 4′-FIU against alphavirus infection *in vivo*, we evaluated 4′-FIU efficacy against MAYV infection in mice (43). WT C57BL/6 mice (*N* = 8 mice/group) were inoculated with 10^3^ PFU of MAYV-CH (44, 45) in the left rear footpad. As for the CHIKV studies, 2 hours post-inoculation mice were treated q.d. with 5 mg/kg of 4′-FIU or vehicle by oral gavage, and at 1, 3, and 5 dpi tissues were collected for viral burden analysis (Fig. 6A). At each time point, 4′-FIU treatment resulted in reduced MAYV burden in most, if not all, tissues evaluated (ipsilateral ankle, contralateral ankle, spleen, ipsilateral wrist, ipsilateral quad, draining popliteal lymph node, and serum) (Fig. 6B through D). These results demonstrate that oral treatment with 4′-FIU limits MAYV infection in mice and supports the idea that this compound has broad-spectrum anti-alphaviral activity.

### Establishment of a treatment window and low efficacious dose for oral treatment of CHIKV infection with 4′-FIU

**Fig 7:**
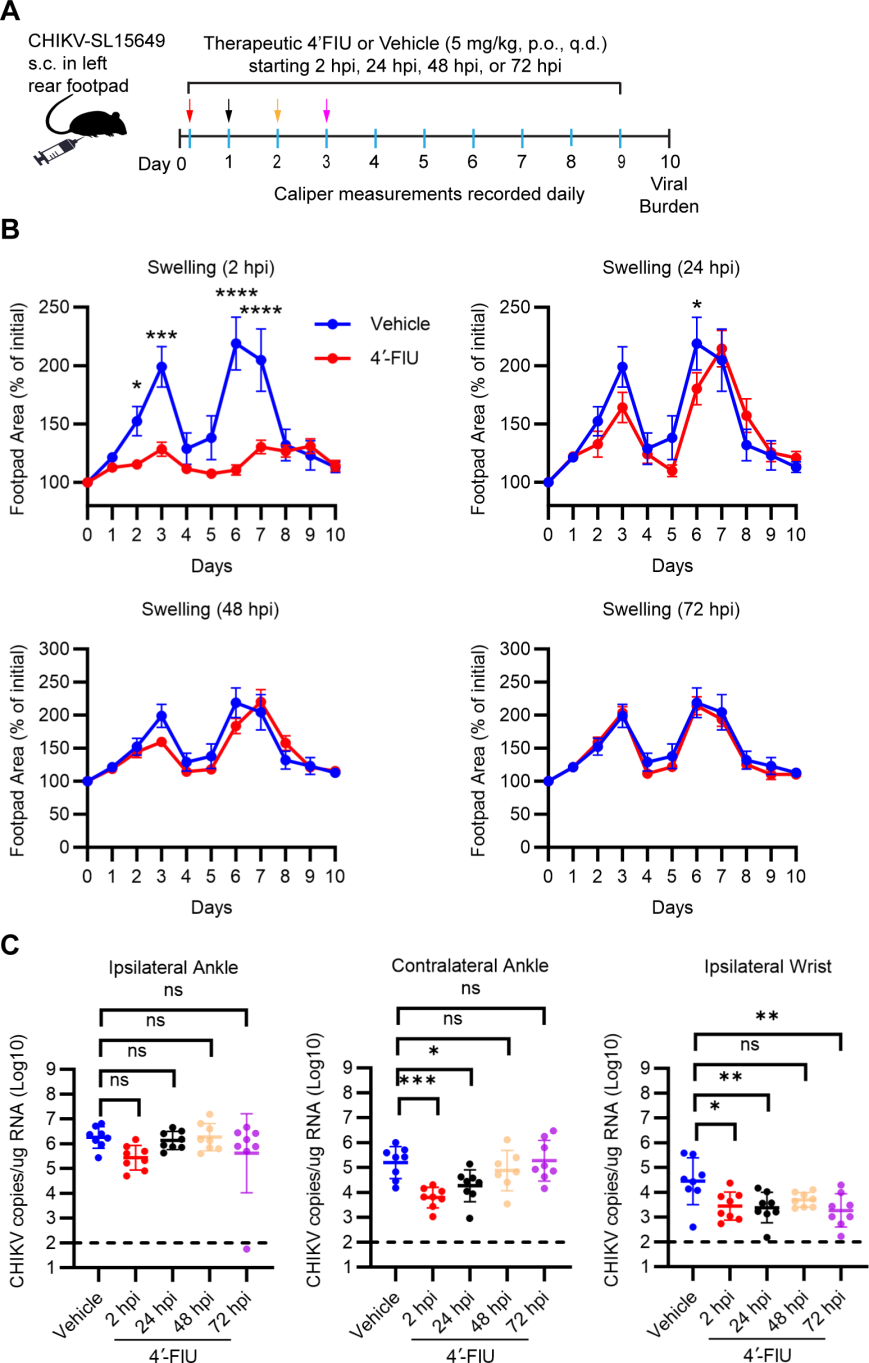
4′-FIU treatment window against acute CHIKV infection in mice. (**A**) Schematic of experimental design generated with BioRender.com. WT C57BL/6 mice (*n* = 8 mice/group) were inoculated with 10^3^ PFU of CHIKV in the left rear footpad. At 2, 24, 48, or 72 hours post-inoculation, mice were treated by oral gavage with vehicle or 5 mg/kg 4′-FIU. Treatment continued once per day until the pre-determined experimental endpoint at 10 days post-virus inoculation. (**B**) Swelling of the ipsilateral foot over time was measured by digital calipers for each treatment condition. (**C**) At 10 dpi, the viral burden in the joint-associated tissues indicated was quantified by RT-qPCR. Statistical significance was determined by repeated measure ANOVA with Tukey’s multiple comparison test (**B**) or one-way ANOVA with Dunnett’s multiple comparison test (**C**). *P < 0.05, *P < 0.05, **P < 0.01, ***P < 0.001, ****P < 0.0001.

Using the CHIKV mouse model, we next determined the therapeutic window for initiation of 4′-FIU treatment (Fig. 7A). As shown in Fig. 7B, initiation of 4′-FIU treatment at 2 hours post-virus inoculation limited CHIKV-induced swelling of the ipsilateral foot and ankle. By contrast, when treatment initiation was delayed until 24 hours, virus-induced swelling was only significantly reduced in 4′-FIU-treated mice at day 6. Although some differences in virus-induced swelling were detected when treatment initiation was delayed until 48–72 hours post-inoculation, none of these differences were statistically significant (Fig. 7B). Because virus-induced swelling can only be measured in tissues near the site of CHIKV inoculation and is driven in part by host immune responses (35, 46, 47), to further evaluate the therapeutic window for treatment of CHIKV infection with 4′-FIU, we measured the viral burden in joint-associated tissues at 10 dpi. In tissues distal to the site of inoculation (contralateral ankle, ipsilateral wrist), the CHIKV burden was significantly reduced in mice treated at 2 or 24 hours post-virus inoculation (Fig. 7C).

**Fig 8:**
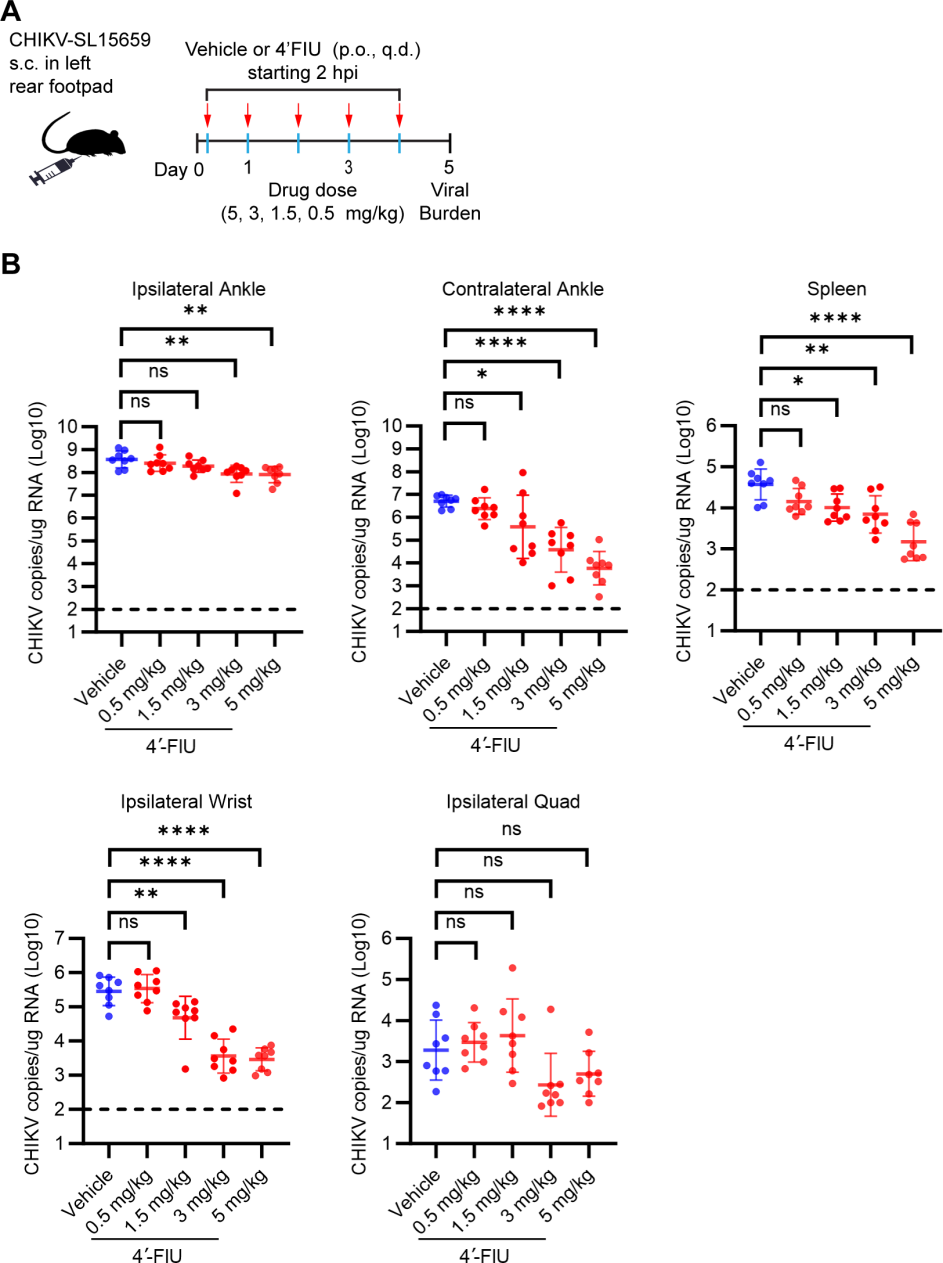
Determination of lowest efficacious dose of 4′-FIU treatment against acute CHIKV infection in mice. (**A**) Schematic of experimental design generated with BioRender.com. WT C57BL/6 mice (*n* = 8 mice/group) were inoculated with 10^3^ PFU of CHIKV in the left rear footpad. At 2 hours post-virus inoculation, mice were treated by oral gavage with vehicle or 5, 3, 1.5, or 0.5 mg/kg 4′-FIU. Treatment continued once per day until the pre-determined experimental endpoint at 5 days post-inoculation. (**B**) At 5 dpi, the viral burden in the tissues indicated was quantified by RT-qPCR. Statistical significance was determined by one-way ANOVA with Dunnett’s multiple comparison test. *P < 0.05, **P < 0.01, ****P < 0.0001.

To establish the lowest efficacious dose of 4′-FIU against CHIKV infection *in vivo*, we compared three dose levels (3, 1.5, and 0.5 mg/kg q.d. by oral gavage) against our established 5 mg/kg dose (Fig. 4), for the capacity to reduce viral tissue burdens at 5 dpi (Fig. 8A). For these studies, treatment was initiated at 2 hours post-CHIKV inoculation. As expected, mice treated with 5 mg/kg displayed reduced viral burdens in tissues both proximal and distal to the site of virus inoculation (Fig. 8B). Mice treated with either 3 mg/kg or 1.5 mg/kg also showed reduced viral tissue burdens, particularly at sites distal to the site of virus inoculation (e.g., contralateral ankle, spleen, and ipsilateral wrist) (Fig. 8B), although 1.5 mg/kg was less effective. We note that the corresponding 4′-FlU-5′-TP tissue exposure levels 24 hours after dosing were lower in animals treated with 3 mg/kg and 1.5 mg/kg compared with 5 mg/kg (Fig. S3), suggesting a minimum threshold of ~0.5–1.0 nmol/g 4′-FlU-5′-TP tissue level for efficacy against CHIKV infection in musculoskeletal tissues.

## DISCUSSION

CHIKV is a re-emerging human pathogen with rapid global spread and represents a serious public health threat (1–3). Other significant human alphavirus pathogens include the arthritogenic alphaviruses MAYV, ONNV, and RRV and the encephalitic alphaviruses Venezuelan, Eastern, and Western equine encephalitis viruses. The absence of approved antiviral drugs targeting CHIKV or other alphaviruses underscores the urgent need for the development of inhibitors. Most antiviral compounds under investigation for alphaviruses including CHIKV are still in the early stages of research and have been primarily evaluated through *in vitro* assays such as virus growth in cell culture. To move such inhibitors forward, more information is needed on their molecular mechanisms and their *in vivo* efficacy in animal models.

Previous studies showed that 4′-FlU has antiviral activity against both negative- and positive-sense RNA viruses, inhibiting replication of the coronavirus SARS-CoV-2, the paramyxovirus RSV, and orthomyxoviruses such as influenza virus (32, 33). In this study, we demonstrated that 4′-FlU inhibits CHIKV infection by blocking RNA replication. Inhibition resulted in reductions in the yield of infectious CHIKV, the expression of virus- or replicon-encoded reporters, and the activity of *trans*-replicase systems. 4′-FlU also inhibited virus production by a panel of re-emerging alphaviruses including MAYV, ONNV, and RRV. EC_50_ values ranged from ~1.2 μM to 3.7 µM for these viruses and were from ~12 to 35 µM for SINV and SFV. Cell viability assays showed that the CC_50_ of 4′-FlU at 24 hours was more than 2,000 µM in U-2 OS cells, resulting in very high selectivity indices. Thus, even for the less sensitive SINV and SFV, inhibition occurred at concentrations far below those causing cytotoxicity. Time of addition studies showed that 4′-FlU had no significant effects on viral entry or late stages of virus production, but strongly inhibited an early step of the CHIKV life cycle. Results from CHIKV replicon and *trans*-replicase assays showed that the compound inhibited viral RNA replication. Notably, the *trans*-replicase assay separated the expression of the nsPs from the observed inhibition of viral RNA production. The differential sensitivity between CHIKV and SFV was observed in both virus production and *trans*-replicase assays and was shown to correlate with the viral source of nsP4, thus providing strong evidence that 4′-FlU acts on the critical nsP4 RdRp activity. Notably, our studies demonstrate that the *trans*-replicase assay offers a safe alternative for the analysis of inhibitors targeting viral RNA replication, and possibly also for the analysis of mechanisms of resistance to antiviral compounds, without a need to use live viruses, their mutants, or chimeric viruses. This could simplify future studies with inhibitors of highly pathogenic alphaviruses, including those listed as select agents.

RdRp assays using purified recombinant polymerase complexes of SARS-CoV-2, RSV, and influenza virus show that 4′-FlU incorporates into the nascent RNA strand and induces premature chain termination (32, 33, 48). This chain termination typically occurs within three to four nucleotides of incorporation of 4′-FlU-5′-TP, and it is hypothesized that termination results from alterations in the secondary structure of the RNA after incorporation (33, 48). The low cytotoxicity of 4′-FlU suggests selective incorporation into viral rather than cellular RNAs.

Our studies in mice demonstrated that daily oral dosing with 4′-FlU decreased viral burden, virus-induced joint tissue swelling, and production of inflammatory markers for CHIKV, and decreased the viral burden for MAYV. 4′-FlU demonstrated effective oral delivery, resulting in sustained tissue exposure of bioactive 4′-FlU-5′-TP in our mouse model. Inhibition was strongest when the drug was administered starting at 2 hpi, but decreased viral titers were observed with treatment initiated at 24 hpi. It is important to note that CHIKV production and disease symptoms in mice have a more rapid onset than disease in humans (49), so it is difficult to extrapolate to a possible treatment window for human CHIKV infection. The *in vivo* effects of 4′-FlU on replication of both CHIKV and MAYV indicate that it is a strong candidate for further studies of its potential as an antiviral treatment for alphavirus disease.

A significant challenge for the development of antiviral drugs against CHIKV and other alphaviruses lies in their potential capacity to mutate and acquire resistance to antiviral therapies. For example, serial *in vitro* passaging of CHIKV in the presence of favipiravir led to resistance through the acquisition of a K291R mutation within the highly conserved F1 motif of the nsP4 protein (27). It is unknown if resistance to 4′-FlU would occur *in vitro* or *in vivo* by CHIKV, or would prove problematic in a clinical setting. Future studies can address these questions by performing full CHIKV sensitivity profiling against 4′-FlU and by analysis of the molecular basis for any observed resistance.

In summary, we here describe the effects of 4′-FlU treatment on alphavirus replication using cell culture studies of virus production, CHIKV replicons, and *trans*-replicase assays, and *in vivo* studies in mouse models of CHIKV and MAYV infection and disease. Together, our results establish 4′-FlU as an orally available drug with efficacy against CHIKV and related alphaviruses.

## MATERIALS AND METHODS

### Cell lines

U-2 OS cells (ATCC, HTB-96) were cultured in modified McCoy’s 5A medium supplemented with 10% fetal bovine serum (FBS). BHK-21 cells (ATCC CCL-10) were grown in α-minimal essential media (Life Technologies) supplemented with 10% FBS, 10% tryptose phosphate broth, and 0.29 mg/mL L-glutamine. Vero cells (ATCC CCL-81) were cultured in DMEM/F-12 (Gibco 11330032) with 10% FBS, 1× nonessential amino acids (Gibco 11140-050), sodium bicarbonate, and 2 mM L-glutamine. Growth media contained 100 U penicillin/mL and 100 µg streptomycin/mL; cells were grown at 37°C in 5% CO_2_.

### CHIKV replicon cell line

The CHIKV replicon was based on the LR2006OPY1 strain. Four mutations enabling continuous replication in human cells (50) were introduced into the ns region (a Phe391 to Leu substitution in nsP1, a Gly-Glu-Glu-Gly-Ser insertion between residues 647 and 648 of nsP2, a Pro718 to Gly substitution in the methyltransferase-like (MTL) domain of nsP2, and an Ile175 to Leu substitution in nsP3). The sequence corresponding to the cleavage site between nsP3 and nsP4 was duplicated as previously described (51) and used for insertion of the sequence encoding a nLuc reporter fused with the PEST sequence. The sequence encoding for puromycin N-acetyltransferase was placed under the control of a subgenomic promoter in the replicon construct. The obtained replicon was designated as CHIKV-Rep-nLuc, and used for *in vitro* transcription and then electroporation of U-2 OS cells. The cells were selected with puromycin (1 mg/mL) starting from 16 h post-electroporation to 14 days.

### Viruses

Virus stocks used *in vitro* assays were produced from the following infectious cDNA clones: CHIKV 181/25 (pSinRep5-181/25ic [52], provided by Dr. Terence S. Dermody), MAYV (MAYV-CH-mKATE), RRV (RRV-T48-ZsGreen), ONNV (ONNV-ZsGreen), SFV (pSP6-SFV4 [53]), and SINV (dsTE12Q [54], provided by Dr. Beth Levine). CHIKV strain SL15649, originally isolated from the serum collected from a patient infected with CHIKV in Sri Lanka in 2006 (35), and MAYV CH, originally isolated from a MAYV-infected patient in Peru in 2001 (44), were derived from the corresponding infectious cDNA clones and used in mouse experiments. The infectious cDNA clone of the CHIKV-nLuc reporter virus (181/25 strain) was generated by the insertion of the nLuc gene behind the natural subgenomic promoter together with an additional subgenomic promoter for the expression of ORF2. PCR amplification was used to generate the additional subgenomic promoter and nLuc, using the following primers: F1-SG-promoter-Swa1: GACCCGCTAAAAAGGTTATTTAAATTGGGCAAACCGTTAG, R1-SG-promoter-Luc: CTTCGAGTGTGAAGACCATTATGGCTGATTGGTATTTAG, F2-SG-promoter-nLuc: CTAAATACCAATCAGCCATAATGGTCTTCACACTCGAAG, R2-nLuc-Spe1: GTCCTCTGAGCTTCACTAGTTTACGCCAGAATGCGTTCGCAC. The PCR fragments were then inserted into the 181/25 infectious clone linearized by digestion with Swa1 and Spe1, using the Gibson assembly kit according to the manufacturer’s instructions (#E2611, NEB).

The infectious cDNA clones were linearized, purified, and used for *in vitro* transcription. Capped RNA transcripts were generated using an SP6 *in vitro* transcription kit (Life Technologies), and electroporated into low-passage BHK-21 cells. Cells were grown at 37°C for 27–30 hours until cytopathic effect (CPE) was present. Virus-containing cell culture supernatants were collected and clarified by centrifugation at 3,000 rpm in the Thermo Scientific TX-1000 rotor for 20 minutes at 4°C. Virus stocks used *in vitro* assays were titered by FFA in U-2 OS cells.

### Focus formation assay

To measure the virus titers from *in vitro* assays, U-2 OS or Vero cells in 96 well-plates were infected with 10-fold serial dilutions of virus in Med-A (minimum essential medium plus 0.2% BSA and 10 mM Hepes) for 2 hours, then overlaid with 1% carboxylmethylcellulose in modified Eagle’s Medium supplemented with 2% FBS and 10 mM HEPES pH 7.4. At 18 hpi, cells were fixed by adding 100 µL warm 1% paraformaldehyde (PFA; Electron Microscopy Science) in PBS to the overlay and incubating for 1 h. After five washes with PBS, the cells were permeabilized with 0.1% saponin in PBS containing 0.1% BSA. The CHIKV-, SFV-, ONNV-, and MAYV-infected cells were incubated with mAb to E2 (E2-1) (53). RRV-infected cells were incubated with rabbit polyclonal antibody against SFV capsid (12). SINV-infected cells were immunostained with mAbs R2 and R6 to SINV E1 and E2 (55). Then, the cells were incubated with horseradish peroxidase-conjugated goat anti-mouse or anti-rabbit IgG. Foci were developed using TrueBlue Peroxidase substrate (#5510-0030, Seracare) and quantified on an ImmunoSpot S6 Macroanalyzer (Cellular Technologies).

To quantify infectious virus from mice, 10-fold serial dilutions of homogenized tissue in DMEM/1% FBS/10 mM HEPES were generated and absorbed onto Vero cells in a 96-well plate for 2 hours. 1% methylcellulose in minimal essential media (MEM) alpha/2% FBS/10 mM HEPES was used to overlay cells and then incubated at 37°C for 18 h. Cells were fixed with 1% PFA and then probed with either CHK-11 monoclonal antibody (56) at 500 ng/mL for CHIKV samples or CHK-48 monoclonal antibody (56) at 250 ng/mL for MAYV samples. Antibodies were diluted in 1× PBS/0.1% saponin/0.1% BSA (Perm Wash). After incubation for 1 hour and repeated washes with PBS, cells were treated with a secondary goat anti-mouse IgG horseradish peroxidase-conjugated antibody diluted 1:2000 in Perm Wash. Foci were visualized and quantitated as above.

### Dose-response antiviral assays *in vitro*

For virus-yield-based dose-response assays, 75,000 U-2 OS cells per well were seeded into 24-well plates and cultured for 24 hours. The cells were infected with the indicated alphaviruses at MOI = 0.1 in Med-A for 1 hour and washed three times with complete medium. Threefold serial dilutions of 4′-FlU were prepared and added. At 24 hpi, the media were clarified by centrifugation and the virus present was quantitated by FFA. For the dose-response assay of CHIKV-nLuc, cells were lysed at 24 hpi, and luciferase activity was measured using Nano-Glo Dual-Luciferase substrate (Promega) and PerkinElmer Victor X5 multilabel plate reader. For dose-response assays of CHIKV-Rep-nLuc, 7,500 cells harboring the replicon were seeded per well in 96-well plates, cultured for 24 h, and treated with threefold serial dilutions of 4′-FlU for 24 h. Then the cells were lysed, and luciferase activity was determined as described for the CHIKV-nLuc infected cells. For all dose-response antiviral assays, cells treated with DMSO were used as negative control. Relative luciferase activity or virus production was normalized with negative control. Dose-response curves were further analyzed by normalized non-linear regression with variable slope to determine 50% EC_50_ and 95% confidence intervals (*CIs*) with Prism 10 (GraphPad).

### Cytotoxicity assays

To measure the effect of 4′-FlU on cellular viability, 75,000 U-2 OS cells per well were seeded into 24-well plates and cultured for 24 hours. The cells were incubated with 2,000 µM, 1,000 µM 4′-FlU, or threefold serial dilution of 4′-FlU from 500 µM for 24 h at 37°C. DMSO was used as a negative control for normalization. Then the cells were incubated with PrestoBlue (ThermoFisher Scientific) for 1 hour at 37°C and fluorescence was measured with the PerkinElmer Victor X5 multilabel plate reader. CC_50_ and 95% CIs were calculated by normalized non-linear regression with variable slope using Prism (GraphPad).

### Time of addition assay *in vitro*

U-2 OS cells (75,000 per well) were seeded into 24-well plates, cultured for 24 hours, and infected with CHIKV at MOI = 3 in Med-A for 1 hour. The cells were washed three times with a complete medium and cultured in 0.5 mL complete medium. 10 µM of 4′-FlU was added in Med-A or complete medium at different time points. At 14 hpi, the medium was clarified by centrifugation and the virus present was quantitated by FFA.

### *trans*-replication assays

Assays utilized plasmids expressing P123 of selected alphaviruses (designated CMV-P123-CHIKV, CMV-P123-MAYV, CMV-P123-SINV, and CMV-P123-SFV) together with plasmids expressing nsP4 of selected alphaviruses (designated CMV-nsP4-CHIKV, CMV-nsP4-MAYV, CMV-nsP4-SINV, and CMV-nsP4-SFV) as previously described (57). Replication-competent RNA templates were expressed using human RNA polymerase I promoter-based plasmids that express Firefly luciferase (Fluc) and *Gaussia* luciferase (Gluc) reporters (designated HSPolI-FG-CHIKV, HSPolI-FG-MAYV, HSPolI-FG-SINV, and HSPolI-FG-SFV) as described previously (58). See also Fig. 3B.

The *trans*-replication assay was carried out using U-2 OS cells grown in a 48-well plate. Approximately 30,000 cells were plated per well, cultured for 24 hours, and then co-transfected with 250 ng plasmid encoding template RNA, 250 ng of plasmid encoding P123, and 190 ng of plasmid encoding nsP4. Transfections were performed using LipoFectamine LTX with PLUS reagent (ThermoFisher Scientific) according to the manufacturer’s instructions. At 4 hours post-transfection, the media were replaced with fresh media containing 4′-FlU at concentrations of 100 µM, 33.3 µM, 11.1 µM, 3.7 µM, 1.2 µM, or 0.4 µM, or DMSO used as vehicle control. Cells were incubated for 20 h at 37°C, lysed and Fluc and Gluc activities were measured using the Dual-Luciferase Reporter Assay System (Promega). Dose-response curves were analyzed by log(inhibitor) vs response (three parameters) to determine 50% EC_50_ with Prism 10 (GraphPad).

### Immunofluorescence microscopy

U-2 OS cells (1.5 × 10^5^ per well) were seeded onto glass coverslips in 6-well plates, cultured for ~24 hours, infected with CHIKV at MOI = 3 for 1 h, and treated with 10 µM 4′-FlU at 0 hpi or 4 hpi. Cells were fixed at 8 hpi, permeabilized, and stained as previously described (59), using a primary antibody to dsRNA (Scicons, J2-1406) and rabbit polyclonal antiserum to nsP4 (in-house). Confocal images were acquired using a 63× oil immersion lens and a Leica TCS SP5 microscope in the Einstein Analytical Imaging Facility.

### Mouse experiments

Four-week-old male and female C57BL/6 mice were obtained from Jackson Laboratories. Eight mice per group (four males and four females) were used for all studies. Mice were anesthetized with isoflurane vapors and inoculated in the left rear footpad with a 10 µL volume of PBS/1% FBS, containing 10^3^ PFU of CHIKV SL15649 or 10^3^ PFU of MAYV-CH. Mice were treated therapeutically with 5 mg/kg 4′-FlU or vehicle (10 mM trisodium citrate in water), by oral gavage (p.o.), once daily (q.d.) starting 2 h post-inoculation. Swelling measurements were taken daily as CHIKV SL15649 infection results in characteristic bi-phasic swelling of the ipsilateral foot and ankle in C57BL/6 mice, which can be used as an indicator of acute CHIKV infection in the mouse model (60). There were no signs of drug toxicity as mice treated with 4′-FIU gained weight consistently throughout the experiment. For 4′-FlU therapeutic window studies, mice were treated therapeutically with 5 mg/kg 4′-FlU or vehicle), p.o., q.d., starting at 2, 24, 48, or 72 hours post-inoculation. For 4′-FlU dose-response studies, mice were treated therapeutically with 0.5, 1.5, 3, or 5 mg/kg 4′-FlU or vehicle, p.o., q.d., starting 2 hours post-inoculation, and euthanized at the experimental endpoint. The tissue distribution profiling of 4′-FlU and 4′-FlU-5′-TP in mice was performed as previously described (33). Animals were treated with 5 mg/kg 4′-FlU, p.o., q.d. Tissue samples were collected at 2 h, 24 h, and 120 h after dosing was initiated and immediately frozen on dry ice. Samples of frozen animal tissue were extracted with cold (4°C) 70% acetonitrile in water by homogenization in a Lysera bead mill outfitted with a cryo cooling unit (Biotage, Uppsala, Sweden). To remove large solids, the homogenate was centrifuged for 5 min at 10,000 rpm in an Eppendorf 5425R centrifuge (Eppendorf, Hamburg, Germany). The supernatant was then transferred to a micro-centrifuge tube and centrifuged again in an Eppendorf 5425R centrifuge for 5 min at 15,000 rpm to remove any remaining solids. The resulting supernatant was transferred to an HPLC vial and analyzed via LCMS-MS according to a qualified, internal standard-based bioanalytical method. HPLC separation was performed on an Agilent 1260 system (Agilent Technologies, Santa Clara, CA, USA) equipped with an autosampler, column oven, UV lamp, and binary pump. A SeQuant ZIC-pHILIC PEEK-coated (100 × 4.6 mm, 5 µm) column (Merck Millipore, Burlington, MA, USA) was used for the separation of 4′-FlU, its 5′-monophosphate metabolite, and its 5′-triphosphate metabolite, 4′-FlU-5′-TP. Mobile phase A consisted of 50 mM ammonium bicarbonate buffer in HPLC grade water pH-adjusted to 9.8, and mobile phase B consisted of pure acetonitrile. An 11-minute isocratic HPLC method was performed to separate the analytes using 33% mobile phase A and 67% mobile phase B. Mass spectrometry analysis was performed on a QTrap 7500 mass spectrometer (Sciex, Framingham, MA, USA) using negative mode electrospray ionization (ESI) in scheduled multiple reaction monitoring (sMRM) mode. Data analysis was performed using SciexOS Software (Sciex, Framingham, MA, USA).

### Virus quantification from mice

Sera were collected from mice, and tissues were collected in 1× PBS/1% FBS/1× Ca^2+^Mg^2+^ for viral burden analysis by FFA. Tissues were homogenized using MP Biomedicals FastPrep-24 Classic (1 cycle of 30 seconds at 4.0 m/s). Alternatively, tissues were collected in TRIzol Reagent (Invitrogen), homogenized using MP Biomedicals FastPrep-24 Classic (1 cycle of 20 seconds at 5.5 m/s), and viral burden analysis was performed by RT-qPCR as outlined below.

### Real-time quantitative polymerase chain reaction

To quantify viral RNA in tissues, RNA was extracted from tissues homogenized in Trizol using a PureLink RNA Mini Kit (Invitrogen). CHIKV or MAYV cDNA was generated from 1 µg of tissue-derived total RNA using random hexamer primers and SuperScript IV reverse transcriptase (Invitrogen). CHIKV copies were quantified using CHIKV-specific forward primer (5′-TTTGCGTGCCACTCTGG-3′) and reverse primer (5′-CGGGTCACCACAAAGTACAA-3′) with an internal TaqMan probe (5′-ACTTGCTTTGATCGCCTTGGTGAGA-3′), all within the nsP2 region of the genome, as previously described (41). MAYV copies were quantified using MAYV-specific forward primer (5′-CACGTCCCCTATACCCAGAC-3′) and reverse primer (5′-GAAACGGGTATGTTGCCGAC-3′) with an internal TaqMan probe (5′-TGGCAAAAAGACAGGGACTCA-3′) all within the E1 region of the viral genome. The total number of CHIKV or MAYV RNA copies was extrapolated from a standard curve generated from samples containing 10^8^ to 10°Copies of CHIKV or MAYV genomic RNA spiked into total RNA from BHK-21 cells, and cDNA was synthesized *in vitro* under conditions identical to those for samples from tissues. Samples were run and analyzed on a QuantStudio 7 Real-Time PCR system (Applied Biosystems).

### Inflammatory cytokine analysis

U-Plex multiplex plate [Meso Scale Discovery U-Plex Biomarker Group 1 (ms) Assay] was coated with mouse analyte-specific antibodies (IFN-γ, IL-1β, TNF-α, MCP-1/CCL2, MIP-1α/CCL3, and MIP-1β/CCL4) and incubated at 4°C overnight. Serum samples were inactivated using 0.1% Triton X, allowing them to be worked with at Biosafety level 2 (BSL2). Serum was diluted 1:2 into Diluent 41. Calibrator standards were prepared using Calibrators 5 and 12 (MSD) and allowed to equilibrate to room temperature. Calibrator standards and samples were loaded onto the U-Plex multiplex plate and incubated at room temperature for 1 hour while shaking at 700 rpm. Detection antibodies were added to the plate and incubated at room temperature for 1 hour while shaking at 700 rpm. MSD Gold Read Buffer was added to the plate and the plate was read on an MSD Discovery Instrument using Methodical Mind software. Read data were transferred into MSD Workbench software for analysis.

### Inflammatory gene expression analysis

To quantify inflammatory gene expression levels in ankle tissue, RNA was extracted from tissues homogenized in Trizol using PureLink RNA Mini Kit (Invitrogen). CHIKV cDNA was generated from 1 µg of tissue-derived total RNA using random hexamer primers and SuperScript IV reverse transcriptase (Invitrogen). Expression levels were quantified by RT-qPCR using gene-specific TaqMan Gene Expression Assays for IFN-γ (Life Technologies, Mm01168134_m1), CCL2 (Life Technologies, Mm0041242_m1), TNF-α (Life Technologies, Mm00443258_m1), and IL-1β (Life Technologies, Mm01336189_m1). 18S ribosomal RNA (Life Technologies, Hs99999901_s1) was used as a normalization control. Samples were run and analyzed on a QuantStudio 7 Real-Time PCR system (Applied Biosystems).

### Statistical analysis

Statistical significance was assessed using Student’s unpaired two-tailed *t*-tests, ordinary one-way ANOVA, or two-way ANOVA with multiple comparisons test in GraphPad Prism, version 10. The number of replicates per experiment and the *P* values are indicated in the figures and legends.

## SUPPLEMENTAL MATERIAL

Supplemental Figures mbio.00420-24-s0001.pdf Figures S1-S3.

ASM does not own the copyrights to Supplemental Material that may be linked to, or accessed through, an article. The authors have granted ASM a non-exclusive, world-wide license to publish the Supplemental Material files. Please contact the corresponding author directly for reuse.
