# Supplementary material for: 4′-Fluorouridine inhibits alphavirus replication and infection in vitro and in vivo
Source: mBio. 2024 May 3;15(6):e00420-24. doi: 10.1128/mbio.00420-24 (PMC11237586; doi:10.1128/mbio.00420-24)
Supplement: Supplemental Figures — Figures S1-S3. [file mbio.00420-24-s0001.pdf]

Supplemental Figures

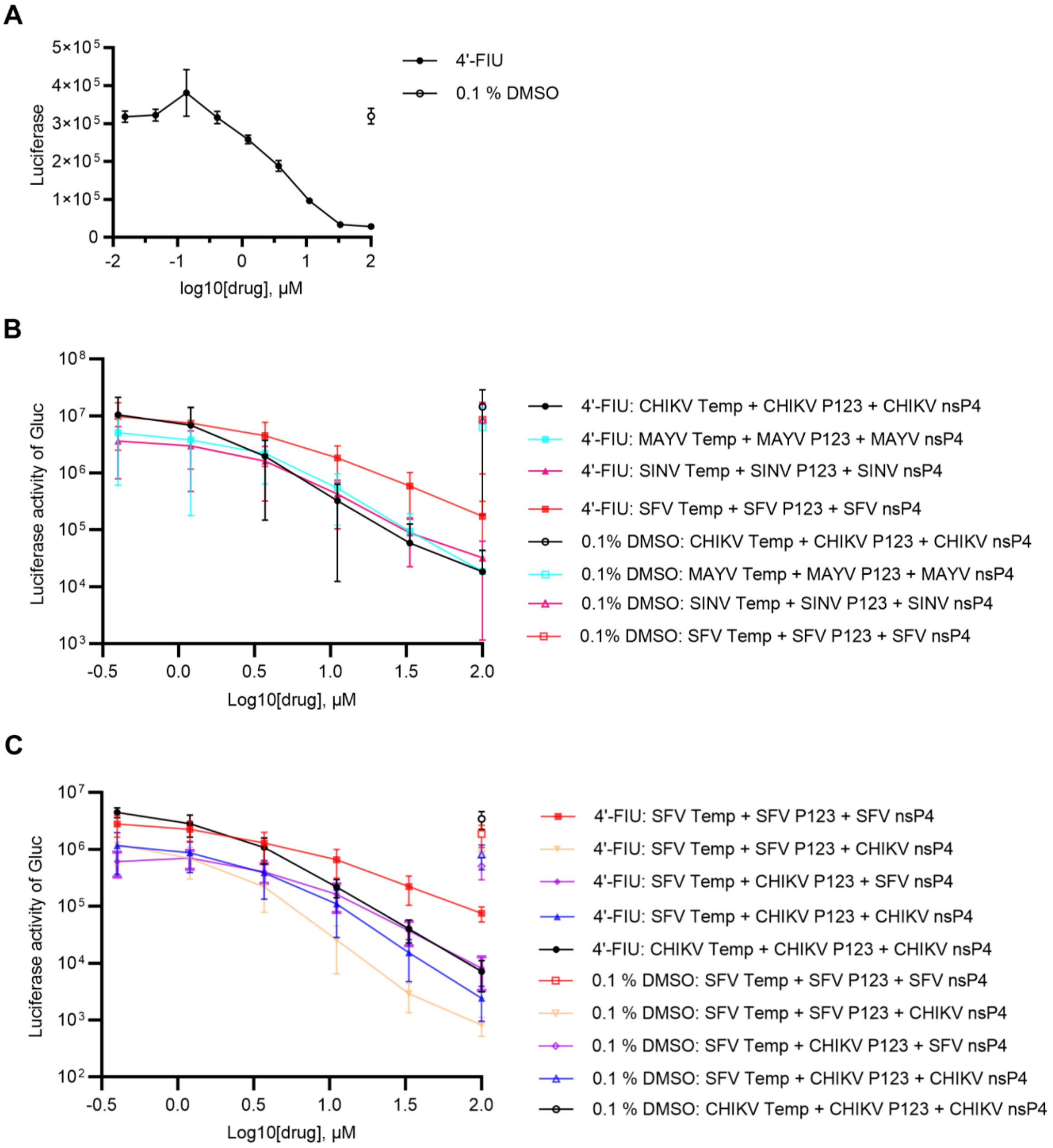

Fig. S1. 4′-FIU blocks alphavirus RNA replication. Figure shows the non-normalized data from the experiments in Fig. 3A, Fig. 3C, and Fig. 3D (here labeled S1A, S1B, and S1C, respectively). Luciferase activity of each reporter is shown in the presence of the indicated concentration of DMSO alone (the highest concentration used as vehicle) or 4′-FIU.

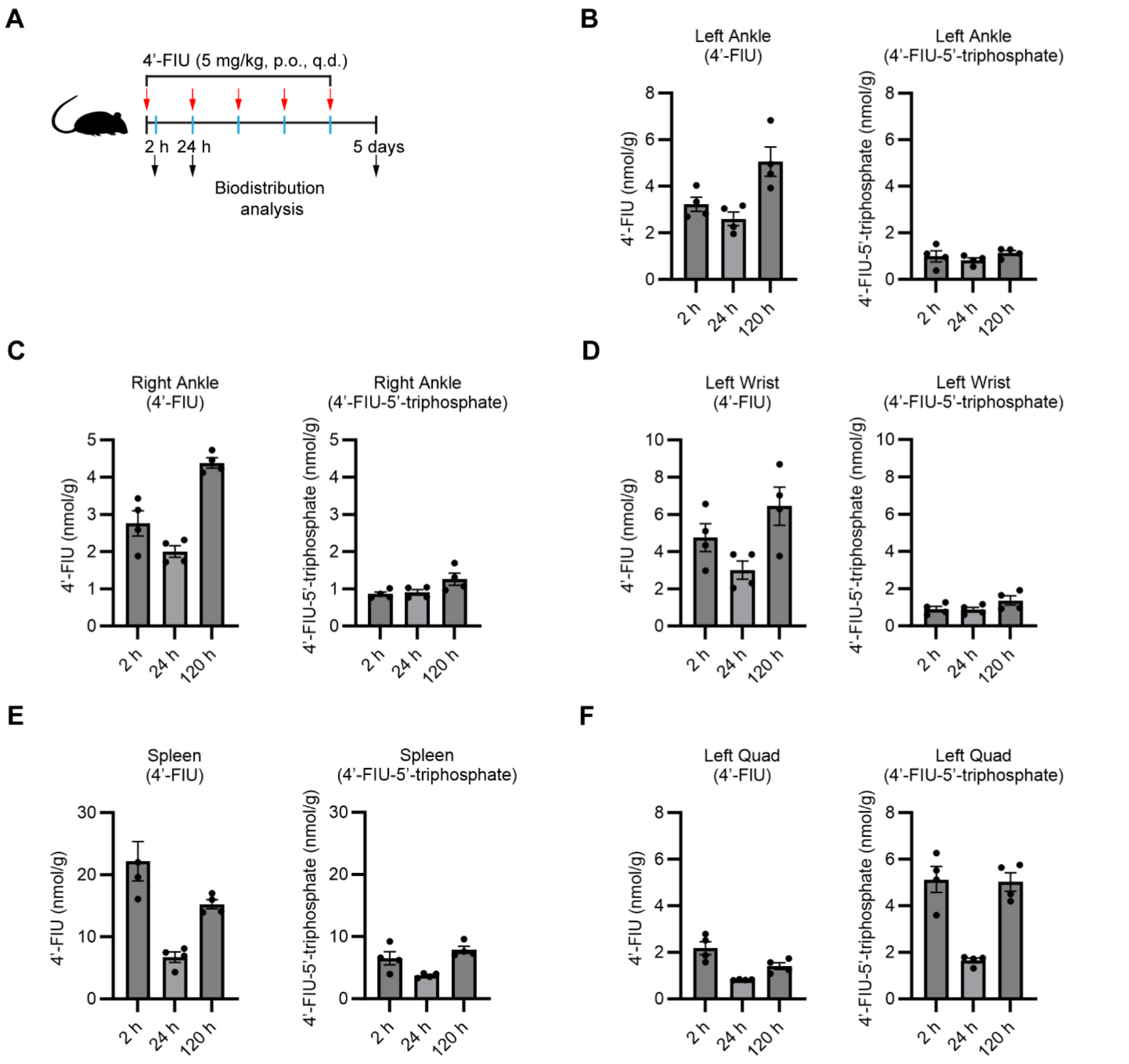

Fig. S2. Biodistribution of 4′-FIU and 4′-FIU-5′-TP. (**A**) Schematic of experimental design created with BioRender.com. WT C57BL/6 mice (n = 4 mice/group) were treated by oral gavage with 5 mg/kg 4′-FIU. Treatment continued once per day until the pre-determined experimental endpoints at 24 h and 120 h post-treatment initiation. (**B-F**) The amount of 4′-FIU and 4′-FIU-5′-TP in the left ankle (**B**), right ankle (**C**), left wrist (**D**), spleen (**E**) and left quad (**F**) was quantitated. Data show the mean ± SD of measurements on 4 mice (shown as points).

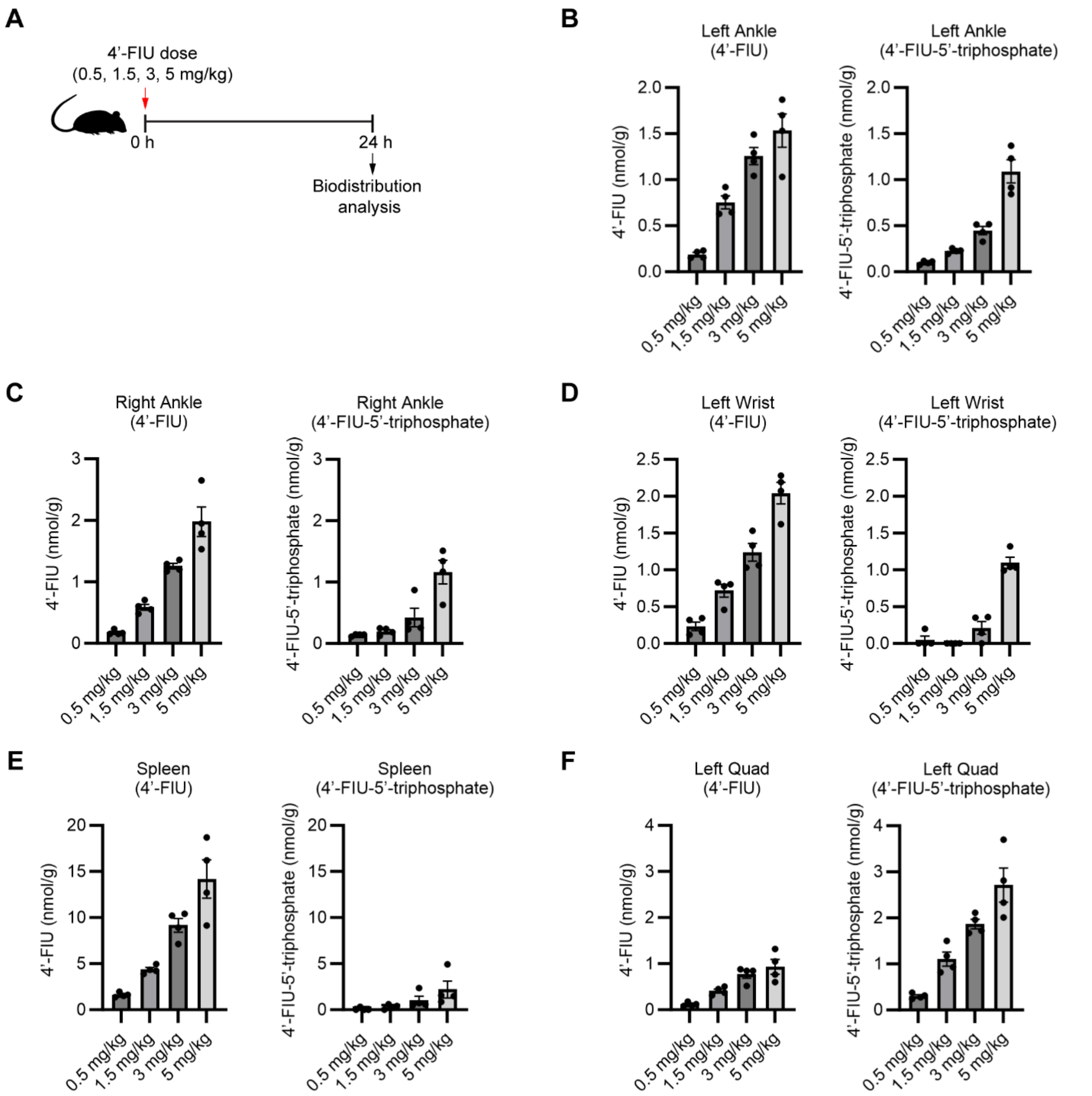

Fig. S3. Tissue distribution of 4′-FIU and 4′-FIU-5′-TP in mice 24 h after a single oral dose of 4′-FIU at different amount. The experimental schematic was generated using BioRender.com. WT C57BL/6 mice (n = 4 mice/group) were treated with 0.5, 1.5, 3, or 5 mg/kg 4′-FIU by oral gavage. (**B-F**) 24 h later, the amount of 4′-FIU and 4′-FIU-5′-TP in the left ankle (**B**), right ankle (**C**), left wrist (**D**), spleen (**E**) and left quad (**F**) was quantitated. Data show the mean ± SD of measurements on 4 mice (shown as points).
